# Methyl 4-(4-hy­droxy­phen­yl)-6-methyl-2-sulfanyl­idene-1,2,3,4-tetra­hydro­pyrimidine-5-carboxyl­ate

**DOI:** 10.1107/S1600536814002888

**Published:** 2014-02-15

**Authors:** Nikhath Fathima, H. Nagarajaiah, Noor Shahina Begum

**Affiliations:** a Department of Studies in Chemistry, Bangalore University, Bangalore 560 001, India

## Abstract

In the title mol­ecule, C_13_H_14_N_2_O_3_S, the di­hydro­pyrimidine ring is in a flattened sofa conformation, with the methine C atom forming the flap. The dihedral angle between the mean plane of the five essentially planar atoms of the di­hydro­pyrimidine ring [maximum deviation = 0.056 (4) Å] and the benzene ring is 89.4 (2)°. The O atom of the carbonyl group is in a *trans* conformation with respect to the C=C bond of the di­hydro­pyrimidine ring. In the crystal, N—H⋯O and O—H⋯S hydrogen bonds connect mol­ecules, forming a two-dimensional network parallel to (001).

## Related literature   

For general background and the biological activity of di­hydro­pyrimidines, see: Kappe (2000 ▶); Jauk *et al.* (2000 ▶); Mayer *et al.* (1999 ▶); For a related structure, see: Liu *et al.* (2008 ▶).
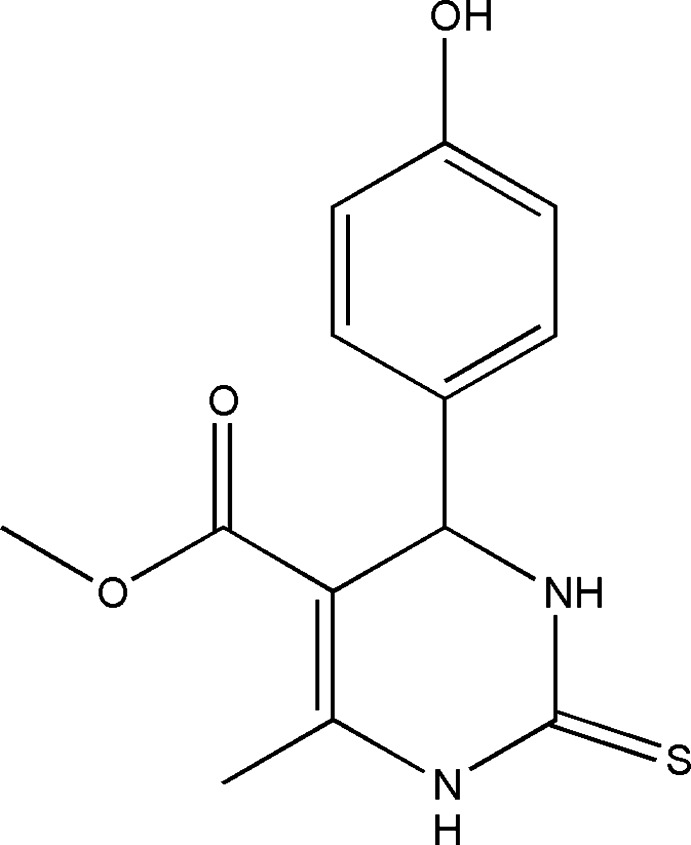


## Experimental   

### 

#### Crystal data  

C_13_H_14_N_2_O_3_S
*M* *_r_* = 278.32
Triclinic, 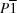

*a* = 7.3016 (12) Å
*b* = 7.6855 (13) Å
*c* = 11.4706 (19) Å
α = 88.337 (3)°
β = 84.870 (3)°
γ = 89.937 (3)°
*V* = 640.84 (18) Å^3^
*Z* = 2
Mo *K*α radiation
μ = 0.26 mm^−1^
*T* = 296 K
0.18 × 0.16 × 0.16 mm

#### Data collection  

Bruker SMART APEX CCD detector diffractometer
Absorption correction: multi-scan (*SADABS*; Bruker, 1998 ▶) *T*_min_ = 0.955, *T*_max_ = 0.960
3897 measured reflections
2703 independent reflections
2084 reflections with *I* > 2σ(*I*)
*R*_int_ = 0.020

#### Refinement  

*R*[*F* ^2^ > 2σ(*F* ^2^)] = 0.067
*wR*(*F* ^2^) = 0.224
*S* = 1.22
2703 reflections
175 parameters
H-atom parameters constrained
Δρ_max_ = 0.76 e Å^−3^
Δρ_min_ = −0.65 e Å^−3^

### 

Data collection: *SMART* (Bruker, 1998 ▶); cell refinement: *SAINT-Plus* (Bruker, 1998 ▶); data reduction: *SAINT-Plus*; program(s) used to solve structure: *SHELXS97* (Sheldrick, 2008 ▶); program(s) used to refine structure: *SHELXL97* (Sheldrick, 2008 ▶); molecular graphics: *ORTEP-3 for Windows* (Farrugia, 2012 ▶) and *PLATON* (Spek, 2009 ▶); software used to prepare material for publication: *WinGX* (Farrugia, 2012 ▶).

## Supplementary Material

Crystal structure: contains datablock(s) global, I. DOI: 10.1107/S1600536814002888/lh5689sup1.cif

Structure factors: contains datablock(s) I. DOI: 10.1107/S1600536814002888/lh5689Isup2.hkl

Supporting information file. DOI: 10.1107/S1600536814002888/lh5689Isup3.cml

CCDC reference:

Additional supporting information:  crystallographic information; 3D view; checkCIF report

## Figures and Tables

**Table 1:** Hydrogen-bond geometry (Å, °)

*D*—H⋯*A*	*D*—H	H⋯*A*	*D*⋯*A*	*D*—H⋯*A*
N1—H1⋯O1^i^	0.86	2.06	2.923 (5)	174
N2—H2⋯O3^ii^	0.86	2.14	2.929 (4)	152
O3—H3⋯S1^iii^	0.82	2.36	3.122 (3)	155

Symmetry codes: (i) 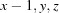; (ii) 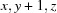; (iii) 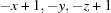.

**Fig. 1.**
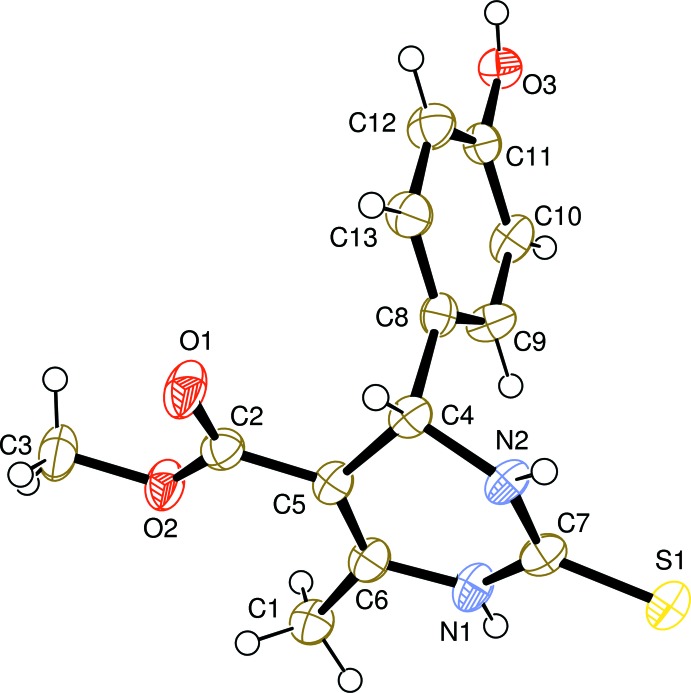
The molecular structure of the title compound. Displacement ellipsoids are drawn at the 50% probability level. H atoms are presented as small spheres of arbitrary radius.

**Fig. 2.**
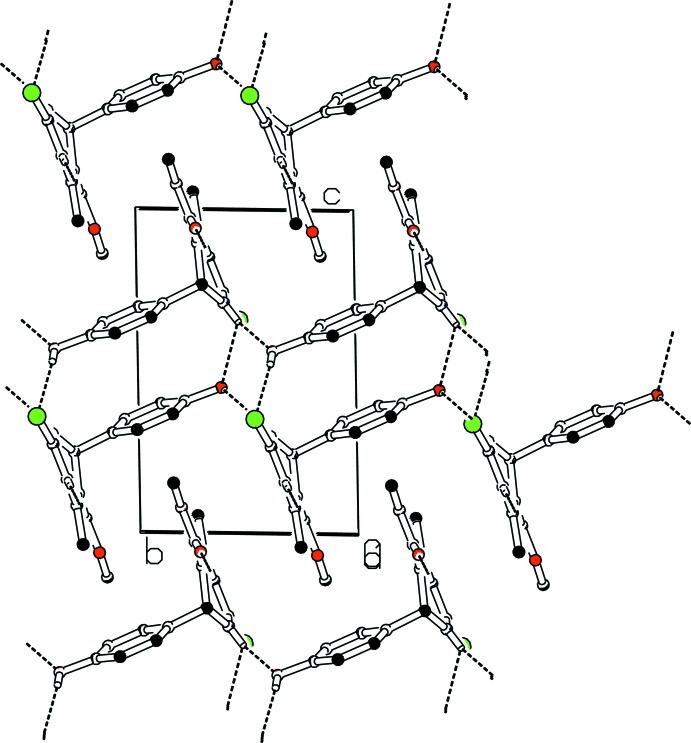
Part of the crystal structure with hydrogen bonds shown as dashed lines.

## supplementary crystallographic information

### 1. Comment

The title compound is a member of the dihydropyrimidines (DHPM) (Kappe, 2000), which have emerged as important target molecules for therapeutic and pharmacological properties such as anticarcinogenic (Mayer *et al.*, 1999) and more recently these compounds have emerged as the integral backbones of several calcium channel modulators (Jauk *et al.*, 2000), anti-hypertensive agents. The title compound was chosen for X-ray diffraction studies with the intention of eliciting structural information which could facilitate further understanding of structural requirements for improved biological activity.

The molecular structure of the title compound is shown in Fig. 1. The dihydropyrimidine ring is in a flattened sofa conformation with atom C4 forming the flap. The dihedral angle between the mean plane of the five essentially planar atoms (N1/N2/C5/C6/C7) of the dihydropyrimidine ring [maximum deviation 0.056 (4) Å for C4] and the benzene ring (C8–C13) is 89.4 (2)°. The oxygen atom of the carbonyl group is in a *trans* configuration with respect to the C5═C6 bond of the dihydropyrimidine ring. In the crystal, N—H···O and O—H···S hydrogen bonds connect molecules forming a two-dimensional network parallel to (001) (Fig. 2).

### 2. Experimental

A mixture of methyl acetoacetate (3.12 g, 25 mmol), 4-hydroxy benzaldehyde (3.02 g, 20 mmol), thiourea (1.83 g, 24 mmol) and LiBr (0.175 g, 2mM) in acetonitrile (25 ml) was heated under reflux for 5 h. After cooling, the reaction mixture was poured onto crushed ice. Stirring was continued for several minutes. The solid product was filtered, washed with cold water, dried and recrystallized from ethanol (yield 85%; m.p.485 K). Single crystals were grown from a chloroform solution of the title compound by slow evaporation at room temperature.

### 3. Refinement

H atoms were placed in calculated positions in the riding-model approximation with N—H = 0.86° A, O—H = 0.82° A, C—H = 0.93, 0.96 and 0.98 Å for aryl, methyl and methine H-atoms respectively, with *U*_iso_(H) = 1.2*U*_eq_(N/C) or 1.5*U*_eq_(C_methyl_/O).

### Crystal data

**Table:**

C_13_H_14_N_2_O_3_S	*Z* = 2
*M**_r_* = 278.32	*F*(000) = 292
Triclinic, *P*1	*D*_x_ = 1.442 Mg m^−^^3^
Hall symbol: -P 1	Mo *K*α radiation, λ = 0.71073 Å
*a* = 7.3016 (12) Å	Cell parameters from 2703 reflections
*b* = 7.6855 (13) Å	θ = 1.8–27.0°
*c* = 11.4706 (19) Å	µ = 0.26 mm^−^^1^
α = 88.337 (3)°	*T* = 296 K
β = 84.870 (3)°	Block, colourless
γ = 89.937 (3)°	0.18 × 0.16 × 0.16 mm
*V* = 640.84 (18) Å^3^	

### Data collection

**Table:**

Bruker SMART APEX CCD detector diffractometer	2703 independent reflections
Radiation source: fine-focus sealed tube	2084 reflections with *I* > 2σ(*I*)
Graphite monochromator	*R*_int_ = 0.020
ω scans	θ_max_ = 27.0°, θ_min_ = 1.8°
Absorption correction: multi-scan (*SADABS*; Bruker, 1998)	*h* = −9→9
*T*_min_ = 0.955, *T*_max_ = 0.960	*k* = −9→9
3897 measured reflections	*l* = −10→14

### Refinement

**Table:**

Refinement on *F*^2^	Primary atom site location: structure-invariant direct methods
Least-squares matrix: full	Secondary atom site location: difference Fourier map
*R*[*F*^2^ > 2σ(*F*^2^)] = 0.067	Hydrogen site location: inferred from neighbouring sites
*wR*(*F*^2^) = 0.224	H-atom parameters constrained
*S* = 1.22	*w* = 1/[σ^2^(*F*_o_^2^) + (0.0743*P*)^2^ + 1.681*P*] where *P* = (*F*_o_^2^ + 2*F*_c_^2^)/3
2703 reflections	(Δ/σ)_max_ < 0.001
175 parameters	Δρ_max_ = 0.76 e Å^−^^3^
0 restraints	Δρ_min_ = −0.65 e Å^−^^3^

### Special details

**Table:**

Geometry. All s.u.'s (except the s.u. in the dihedral angle between two l.s. planes) are estimated using the full covariance matrix. The cell s.u.'s are taken into account individually in the estimation of s.u.'s in distances, angles and torsion angles; correlations between s.u.'s in cell parameters are only used when they are defined by crystal symmetry. An approximate (isotropic) treatment of cell s.u.'s is used for estimating s.u.'s involving l.s. planes.
Refinement. Refinement of *F*^2^ against ALL reflections. The weighted *R*-factor *wR* and goodness of fit *S* are based on *F*^2^, conventional *R*-factors *R* are based on *F*, with *F* set to zero for negative *F*^2^. The threshold expression of *F*^2^ > 2σ(*F*^2^) is used only for calculating *R*-factors(gt) *etc*. and is not relevant to the choice of reflections for refinement. *R*-factors based on *F*^2^ are statistically about twice as large as those based on *F*, and *R*- factors based on ALL data will be even larger.

### Fractional atomic coordinates and isotropic or equivalent isotropic displacement parameters (Å^2^)

**Table:**

	*x*	*y*	*z*	*U*_iso_*/*U*_eq_	
C1	0.3658 (6)	0.2646 (6)	−0.0479 (4)	0.0338 (9)	
H1A	0.4523	0.3220	−0.1042	0.051*	
H1B	0.2455	0.3127	−0.0541	0.051*	
H1C	0.3637	0.1424	−0.0627	0.051*	
C2	0.7615 (6)	0.2492 (5)	0.0356 (4)	0.0304 (9)	
C3	0.9034 (6)	0.1552 (8)	−0.1446 (4)	0.0438 (12)	
H3A	0.9746	0.2601	−0.1569	0.066*	
H3B	0.8711	0.1151	−0.2186	0.066*	
H3C	0.9744	0.0675	−0.1079	0.066*	
C4	0.6180 (5)	0.2981 (5)	0.2393 (4)	0.0278 (8)	
H4	0.7320	0.3627	0.2462	0.033*	
C5	0.5918 (5)	0.2811 (5)	0.1099 (4)	0.0270 (8)	
C6	0.4216 (5)	0.2909 (5)	0.0729 (4)	0.0299 (9)	
C7	0.2938 (6)	0.3954 (5)	0.2612 (4)	0.0294 (9)	
C8	0.6324 (5)	0.1204 (5)	0.3004 (3)	0.0259 (8)	
C9	0.4829 (6)	0.0075 (5)	0.3100 (4)	0.0331 (10)	
H9	0.3732	0.0437	0.2818	0.040*	
C10	0.4930 (5)	−0.1559 (5)	0.3601 (4)	0.0331 (10)	
H10	0.3905	−0.2284	0.3665	0.040*	
C11	0.6558 (5)	−0.2129 (5)	0.4010 (3)	0.0280 (8)	
C12	0.8074 (6)	−0.1033 (6)	0.3943 (4)	0.0365 (10)	
H12	0.9166	−0.1396	0.4232	0.044*	
C13	0.7924 (6)	0.0628 (6)	0.3433 (4)	0.0335 (9)	
H13	0.8938	0.1367	0.3381	0.040*	
N1	0.2744 (5)	0.3334 (5)	0.1523 (3)	0.0313 (8)	
H1	0.1650	0.3200	0.1318	0.038*	
N2	0.4641 (4)	0.3976 (4)	0.2940 (3)	0.0265 (7)	
H2	0.4851	0.4620	0.3514	0.032*	
O1	0.9147 (4)	0.2737 (5)	0.0673 (3)	0.0431 (8)	
O2	0.7382 (4)	0.1894 (4)	−0.0701 (3)	0.0372 (8)	
O3	0.6613 (4)	−0.3807 (4)	0.4445 (3)	0.0308 (7)	
H3	0.7489	−0.3919	0.4840	0.046*	
S1	0.10843 (14)	0.46706 (14)	0.34469 (10)	0.0335 (3)	

### Atomic displacement parameters (Å^2^)

**Table:**

	*U*^11^	*U*^22^	*U*^33^	*U*^12^	*U*^13^	*U*^23^
C1	0.028 (2)	0.039 (2)	0.035 (2)	−0.0028 (17)	−0.0073 (17)	−0.0051 (18)
C2	0.027 (2)	0.030 (2)	0.034 (2)	0.0000 (16)	−0.0038 (16)	0.0000 (17)
C3	0.028 (2)	0.071 (4)	0.031 (2)	0.000 (2)	0.0023 (18)	−0.010 (2)
C4	0.0247 (19)	0.025 (2)	0.034 (2)	−0.0010 (15)	−0.0027 (15)	−0.0020 (16)
C5	0.0235 (19)	0.025 (2)	0.032 (2)	−0.0022 (15)	−0.0016 (15)	−0.0039 (16)
C6	0.0227 (19)	0.032 (2)	0.035 (2)	−0.0038 (15)	−0.0033 (16)	−0.0035 (17)
C7	0.029 (2)	0.0203 (19)	0.039 (2)	0.0006 (15)	−0.0050 (17)	−0.0053 (16)
C8	0.0261 (19)	0.026 (2)	0.026 (2)	−0.0014 (15)	−0.0001 (15)	−0.0064 (15)
C9	0.027 (2)	0.026 (2)	0.047 (3)	0.0016 (16)	−0.0090 (18)	0.0005 (18)
C10	0.0230 (19)	0.029 (2)	0.048 (3)	−0.0071 (16)	−0.0010 (17)	−0.0092 (18)
C11	0.029 (2)	0.029 (2)	0.026 (2)	−0.0008 (16)	−0.0013 (15)	−0.0019 (16)
C12	0.028 (2)	0.036 (2)	0.045 (3)	−0.0038 (17)	−0.0042 (18)	0.0016 (19)
C13	0.027 (2)	0.036 (2)	0.038 (2)	−0.0079 (17)	−0.0044 (17)	0.0040 (18)
N1	0.0225 (17)	0.0339 (19)	0.038 (2)	−0.0018 (14)	−0.0019 (14)	−0.0116 (15)
N2	0.0163 (15)	0.0261 (17)	0.038 (2)	−0.0022 (12)	−0.0056 (13)	−0.0087 (14)
O1	0.0195 (14)	0.071 (2)	0.0393 (19)	−0.0030 (14)	−0.0015 (12)	−0.0107 (16)
O2	0.0239 (15)	0.056 (2)	0.0317 (17)	−0.0016 (13)	−0.0015 (12)	−0.0081 (14)
O3	0.0323 (16)	0.0258 (15)	0.0347 (17)	−0.0018 (12)	−0.0045 (12)	0.0000 (12)
S1	0.0259 (5)	0.0338 (6)	0.0405 (7)	0.0020 (4)	−0.0001 (4)	−0.0079 (4)

### Geometric parameters (Å, º)

**Table:**

C1—C6	1.498 (6)	C7—N2	1.331 (5)
C1—H1A	0.9600	C7—N1	1.370 (5)
C1—H1B	0.9600	C7—S1	1.688 (4)
C1—H1C	0.9600	C8—C13	1.376 (6)
C2—O1	1.223 (5)	C8—C9	1.390 (5)
C2—O2	1.334 (5)	C9—C10	1.370 (6)
C2—C5	1.465 (6)	C9—H9	0.9300
C3—O2	1.443 (5)	C10—C11	1.382 (6)
C3—H3A	0.9600	C10—H10	0.9300
C3—H3B	0.9600	C11—O3	1.371 (5)
C3—H3C	0.9600	C11—C12	1.387 (6)
C4—N2	1.463 (5)	C12—C13	1.398 (6)
C4—C5	1.523 (6)	C12—H12	0.9300
C4—C8	1.525 (6)	C13—H13	0.9300
C4—H4	0.9800	N1—H1	0.8600
C5—C6	1.350 (5)	N2—H2	0.8600
C6—N1	1.390 (5)	O3—H3	0.8200
			
C6—C1—H1A	109.5	N2—C7—S1	123.7 (3)
C6—C1—H1B	109.5	N1—C7—S1	120.2 (3)
H1A—C1—H1B	109.5	C13—C8—C9	117.7 (4)
C6—C1—H1C	109.5	C13—C8—C4	122.3 (3)
H1A—C1—H1C	109.5	C9—C8—C4	120.0 (4)
H1B—C1—H1C	109.5	C10—C9—C8	121.7 (4)
O1—C2—O2	121.7 (4)	C10—C9—H9	119.2
O1—C2—C5	123.1 (4)	C8—C9—H9	119.2
O2—C2—C5	115.2 (3)	C9—C10—C11	120.0 (4)
O2—C3—H3A	109.5	C9—C10—H10	120.0
O2—C3—H3B	109.5	C11—C10—H10	120.0
H3A—C3—H3B	109.5	O3—C11—C10	117.5 (4)
O2—C3—H3C	109.5	O3—C11—C12	122.3 (4)
H3A—C3—H3C	109.5	C10—C11—C12	120.1 (4)
H3B—C3—H3C	109.5	C11—C12—C13	118.5 (4)
N2—C4—C5	108.7 (3)	C11—C12—H12	120.7
N2—C4—C8	110.9 (3)	C13—C12—H12	120.7
C5—C4—C8	111.5 (3)	C8—C13—C12	122.0 (4)
N2—C4—H4	108.6	C8—C13—H13	119.0
C5—C4—H4	108.6	C12—C13—H13	119.0
C8—C4—H4	108.6	C7—N1—C6	123.7 (3)
C6—C5—C2	125.5 (4)	C7—N1—H1	118.1
C6—C5—C4	120.0 (4)	C6—N1—H1	118.1
C2—C5—C4	114.5 (3)	C7—N2—C4	124.6 (3)
C5—C6—N1	119.0 (4)	C7—N2—H2	117.7
C5—C6—C1	128.1 (4)	C4—N2—H2	117.7
N1—C6—C1	112.8 (3)	C2—O2—C3	116.3 (3)
N2—C7—N1	116.2 (4)	C11—O3—H3	109.5
			
O1—C2—C5—C6	165.7 (4)	C8—C9—C10—C11	−1.0 (7)
O2—C2—C5—C6	−14.8 (6)	C9—C10—C11—O3	−176.6 (4)
O1—C2—C5—C4	−16.3 (6)	C9—C10—C11—C12	1.8 (7)
O2—C2—C5—C4	163.2 (3)	O3—C11—C12—C13	176.8 (4)
N2—C4—C5—C6	−25.1 (5)	C10—C11—C12—C13	−1.5 (7)
C8—C4—C5—C6	97.4 (4)	C9—C8—C13—C12	0.4 (7)
N2—C4—C5—C2	156.8 (3)	C4—C8—C13—C12	−177.0 (4)
C8—C4—C5—C2	−80.7 (4)	C11—C12—C13—C8	0.4 (7)
C2—C5—C6—N1	−175.8 (4)	N2—C7—N1—C6	−6.5 (6)
C4—C5—C6—N1	6.3 (6)	S1—C7—N1—C6	172.3 (3)
C2—C5—C6—C1	2.8 (7)	C5—C6—N1—C7	11.6 (6)
C4—C5—C6—C1	−175.1 (4)	C1—C6—N1—C7	−167.2 (4)
N2—C4—C8—C13	−126.2 (4)	N1—C7—N2—C4	−17.7 (6)
C5—C4—C8—C13	112.5 (4)	S1—C7—N2—C4	163.6 (3)
N2—C4—C8—C9	56.5 (5)	C5—C4—N2—C7	32.0 (5)
C5—C4—C8—C9	−64.8 (5)	C8—C4—N2—C7	−90.9 (5)
C13—C8—C9—C10	−0.1 (7)	O1—C2—O2—C3	0.1 (6)
C4—C8—C9—C10	177.3 (4)	C5—C2—O2—C3	−179.4 (4)

### Hydrogen-bond geometry (Å, º)

**Table:**

*D*—H···*A*	*D*—H	H···*A*	*D*···*A*	*D*—H···*A*
N1—H1···O1^i^	0.86	2.06	2.923 (5)	174
N2—H2···O3^ii^	0.86	2.14	2.929 (4)	152
O3—H3···S1^iii^	0.82	2.36	3.122 (3)	155

Symmetry codes: (i) *x*−1, *y*, *z*; (ii) *x*, *y*+1, *z*; (iii) −*x*+1, −*y*, −*z*+1.
